# Prediction of morbidity and mortality in patients with type 2 diabetes

**DOI:** 10.7717/peerj.87

**Published:** 2013-06-11

**Authors:** Brian J. Wells, Rachel Roth, Amy S. Nowacki, Susana Arrigain, Changhong Yu, Wayne A. Rosenkrans, Michael W. Kattan

**Affiliations:** 1 Department of Quantitative Health Sciences, Cleveland Clinic, Cleveland, OH, United States; 2 Department of Family Medicine, Swedish Hospital, Seattle, WA, United States; 3 Center for Biomedical Innovation, Personalized Medicine Coalition, Massachusetts Institute of Technology, Cambridge, MA, United States

**Keywords:** Type 2 diabetes mellitus, Prediction, Propensity, Coronary heart disease, Heart failure, Stroke, Mortality, Electronic health record, Hypoglycemic agents

## Abstract

**Introduction.** The objective of this study was to create a tool that accurately predicts the risk of morbidity and mortality in patients with type 2 diabetes according to an oral hypoglycemic agent.

**Materials and Methods.** The model was based on a cohort of 33,067 patients with type 2 diabetes who were prescribed a single oral hypoglycemic agent at the Cleveland Clinic between 1998 and 2006. Competing risk regression models were created for coronary heart disease (CHD), heart failure, and stroke, while a Cox regression model was created for mortality. Propensity scores were used to account for possible treatment bias. A prediction tool was created and internally validated using tenfold cross-validation. The results were compared to a Framingham model and a model based on the United Kingdom Prospective Diabetes Study (UKPDS) for CHD and stroke, respectively.

**Results and Discussion.** Median follow-up for the mortality outcome was 769 days. The numbers of patients experiencing events were as follows: CHD (3062), heart failure (1408), stroke (1451), and mortality (3661). The prediction tools demonstrated the following concordance indices (c-statistics) for the specific outcomes: CHD (0.730), heart failure (0.753), stroke (0.688), and mortality (0.719). The prediction tool was superior to the Framingham model at predicting CHD and was at least as accurate as the UKPDS model at predicting stroke.

**Conclusions.** We created an accurate tool for predicting the risk of stroke, coronary heart disease, heart failure, and death in patients with type 2 diabetes. The calculator is available online at http://rcalc.ccf.org under the heading “Type 2 Diabetes” and entitled, “Predicting 5-Year Morbidity and Mortality.” This may be a valuable tool to aid the clinician’s choice of an oral hypoglycemic, to better inform patients, and to motivate dialogue between physician and patient.

## Introduction

Optimizing treatment of type 2 diabetes requires the consideration of a number of important outcomes such as vascular morbidity, heart failure, and mortality. Informed treatment decisions are extremely difficult to make because the literature is frequently inconclusive about the preferred treatment. The best outcomes are not always experienced by patients with the best glycemic control (Abraira et al., 1997; Mellbin et al., 2008; Duckworth et al., 2009). And, head-to-head comparisons of clinical outcomes between the oral hypoglycemic agents are lacking (Bolen et al., 2007). In addition, the most effective oral hypoglycemic drug likely depends on patient characteristics and comorbid conditions.

Calculators modeled from cohorts that are not exclusively comprised of patients with type 2 diabetes have been shown to predict poorly when applied to patients with diabetes (Sheridan, Pignone & Mulrow, 2003; Guzder et al., 2005; Coleman et al., 2007). The calculators that have been fashioned specifically for patients with type 2 diabetes only address single outcomes such as stroke (Wolf et al., 1991; Kothari et al., 2002), coronary heart disease (Wilson et al., 1998; Stevens et al., 2001; Lee et al., 2006), or mortality (Hong Kong Diabetes Registry et al., 2008; Wells et al., 2008). Other complications – like heart failure, which is much more prevalent among diabetic patients (12%) than the general population (3.9%) (Nichols et al., 2001)– have not been modeled with respect to risk at all. Each of these risk assessments exist in different locations and were modeled using slightly different populations, parameters and methods. The result is that they are difficult to combine, and not readily used by physicians.

The purpose of this project was to create a tool that would assist a clinician (and patient) with the selection of an oral hypoglycemic medication. The tool was designed to allow the consideration of a number of common complications and mortality separately but simultaneously, in a more realistic approximation of the complex decision-making process of risk optimization.

## Materials and Methods

A retrospective cohort gathered from the electronic health records (EHR) at the Cleveland Clinic between 1998 and 2006 was used for this study. The data were recorded for patients with type 2 diabetes for clinical and administrative purposes. The research in this project was approved by the Institutional Review Board of the Cleveland Clinic Foundation (Study #06-635) which granted a waiver of informed consent. Patients entered and exited the cohort according to their actual clinical courses and had varying follow-up periods. Baseline was defined as the date of first prescription of an oral hypoglycemic agent in an eligible patient.

Patients were included in the analysis and modeling if they were 18 years of age or older, and carried a diagnosis of type 2 diabetes as determined by International Classification of Diseases, 9th revision (ICD-9) codes (250–250.99, 357.2, 362.01, 362.02, 366.41). The diagnosis of diabetes was required to be recorded a minimum of twice in order to reduce the chance of misclassification due to “rule out” diagnoses. Patients on insulin were included in the study since this medication is frequently used in the treatment of type 2 diabetes. However patients on insulin were still required to be on at least one oral hypoglycemic agent in order to exclude patients with type 1 diabetes.

The analysis was limited to patients who were prescribed a single one of the following oral hypoglycemic agents: sulfonylureas (SFUs), meglitinides (MEGs), biguanides (BIGs), or thiazolidinediones (TZDs). Patients with prescriptions for multiple oral agents at baseline were excluded because of the substantial number of possible two- and three-drug combinations. Patients prescribed alpha-glucosidase inhibitors, non-insulin injectable medications, and other less-commonly used medications were excluded because of inadequate sample sizes.

Patients with advanced disease on dialysis were excluded. Those patients who previously experienced the event of interest (e.g., stroke in the stroke model) were excluded from modeling of that specific outcome. Patients with a documented history of a transient ischemic attack were also excluded from the stroke analysis. Some patients with polycystic ovarian syndrome (ICD-9 256.4) can be placed on BIGs for management; to avoid confusion, patients with such a recorded diagnosis were also excluded. The EHR contained 49,939 patients with type 2 diabetes who were prescribed at least one oral agent. The exclusion of 16,872 patients on multiple oral agents left a final sample size of 33,067.

Four primary outcomes were modeled separately: stroke, heart failure, coronary heart disease (CHD), and mortality. Stroke was defined according to ICD-9 codes 430-434 and 436-438. These codes exclude transient ischemic attack as this diagnosis is difficult to capture reliably in the EHR. CHD was defined as a recorded diagnosis of CHD (ICD9 410-414), documentation of a coronary revascularization procedure, or documentation of CHD as a cause of death on death certificate data obtained from the National Death Index (National Center for Health Statistics, Hyattsville, MD). Heart failure was defined as documented heart failure (ICD9 402.01, 402.11, 402.91, 428.00–428.99, 404.01, 404.11, or 404.91) and/or left ventricular ejection fraction (LVEF) >40% on echocardiogram. Mortality was determined from vital status in the electronic health records (EHR) and/or the Social Security Death Index (SSDI).

Potential predictor variables for each of the four models were chosen individually based on their clinical and physiological relevance to the individual outcomes (Table 2). Values for each predictor variable were extracted from the EHR. The baseline value for each predictor variable was defined as the value of the variable on the baseline date. If missing, the most recent historical value was used, or the value closest to the baseline date up to 21 days following baseline. Patients were considered to have a new diagnosis of type 2 diabetes if they had been seen before their baseline date by either an endocrinologist or primary care physician at Cleveland Clinic and did not have a diagnosis of diabetes entered in the EHR at that time.

The analytic dataset was built using SAS, version 9.1. In order to maximize the available information and to reduce the potential bias introduced by deleting incomplete records, missing values were imputed using the Multiple Imputation by Chained Equations (MICE) package, version 2.3, for R. Ten complete imputed datasets were created using predictive mean matching, logistic regression, and polytomous regression for numeric, binary, and categorical variables, respectively. The missing data were imputed using all of the other covariates as well as the outcomes as predictor variables which has been shown to improve accuracy and decrease bias (Moons et al., 2006). Patients were censored at the time of their last follow up (or the date of the last SSDI update for mortality) and therefore imputation was not employed for the outcome information.

For mortality, a Cox proportional hazards regression model was fit with time to death as the outcome. For stroke, heart failure, and coronary heart disease, a competing risks regression model was fit with death considered the only competing event. Statistical analyses were performed using R, version 2.10.

Given that oral hypoglycemic medication is our variable of interest, a limited number of potential interactions were considered that might result from inclusion of medication in the predictive model. An interaction of *medication class x glomerular filtration rate (GFR)* and *medication class x age* was considered due to precautions advised for use of biguanides (BIGs) in older adults and in patients with renal dysfunction. Similarly, for *medication class x congestive heart failure (CHF)*, there are precautions advised for using TZDs and BIGs in patients with heart failure. (This interaction was not included in the heart failure model.) For parsimony, interactions were only included in the final model if they were statistically significant (*p* < 0.05).

A modified version of Harrell’s “model approximation” (aka step-down) method (Harrell, Lee & Mark, 1996) that maximized the concordance index (c-statistic, a measure of predictive discrimination) and not R-squared (a measure of explained variation) was used for variable selection. Variables in the full models for each outcome were chosen according to clinical relevance (Table 2). Medication, as our primary variable of interest, was forced into each model. Interactions were included only when the interaction variables themselves remained in the model. The final model represents the subset of variables maximizing the c-statistic.

Propensity regression was utilized to adjust for residual confounding by indication. There was agreement among the physicians and investigators that this effect was likely to be small between groups placed on SFUs, TZDs, and MEGs, but large when comparing these to the group of patients placed on a BIG alone (i.e. healther patients with less severe disease are more likely to be prescribed BIG). The propensity parameter included in the final regression model was the probability of receiving BIG and was calculated from a logistic regression model that included all other dependent variables. Model accuracy was assessed using ten-fold cross-validation in order to prevent overfit bias. The cross-validation was performed by randomly dividing the dataset into ten equal sections and setting aside one section as a test dataset while using the other nine sections as a training dataset. The variable selection, propensity score calculations, and model building were all performed in the training dataset. The prediction accuracy was assessed in the test dataset that consisted of patients systematically not included in the training data. This process was repeated a total of ten times with each section of the data serving as a test dataset exactly once. The c-statistic was calculated for each model to demonstrate the model’s ability to identify the patient at higher risk (discrimination). Calibration was assessed graphically by plotting the predicted risk against the actual risk in each quintile.

The final prediction model for CHD was compared head-to-head with the Framingham model described by Wilson et al. (1998). This comparison was performed in a subset of patients between 30–74 years of age in order to fairly represent the population for which the Framingham model was intended. In addition, the test dataset was limited to patients for which complete data prior to imputation was available for calculating the Framingham risk score. The final comparison dataset after these restrictions consisted of 7,714 patients. The Framingham model was designed to produce 10-year risk, whereas our model produces 5-year risk predictions. Thus, an assumption was made that the Framingham model follows an exponential association, and the 5 year risk was estimated accordingly. However, since these models are not time-dependent, the particular predicted follow up time will have no effect on the calculated discrimination (a patient with higher risk in 5 years will also have higher risk in 10 years). The predictions used to compare with the Framingham model were derived from the ten-fold cross validation and were therefore “overfit- corrected” as none of the predictions were made on patients used to build the model.

**Figure 1:**
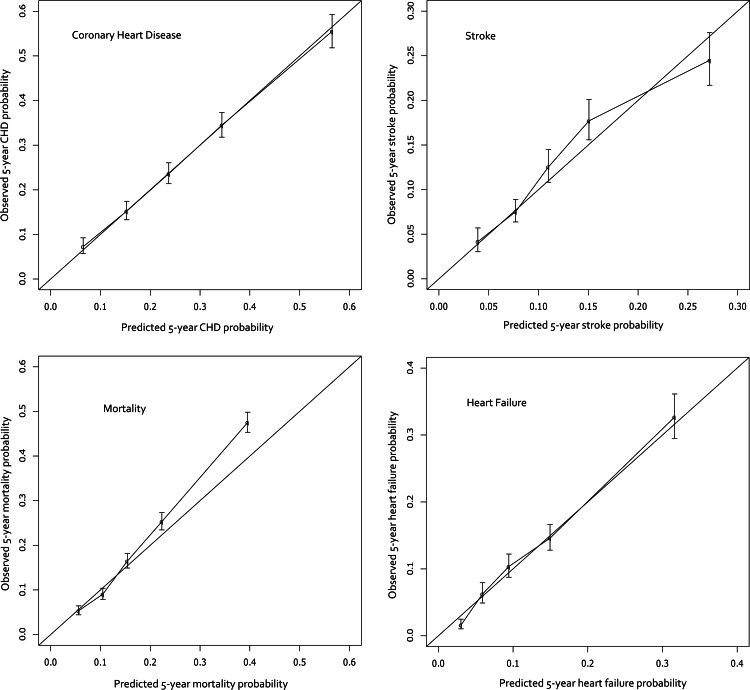
Calibration curves for the final models. The curves display the predicted probabilities on the *x*-axis and the Kaplan–Meier estimations on the *y*-axis according to quintiles of the predicted probabilities.

The final prediction model for stroke is compared to that created by the United Kingdom Prospective Diabetes Study (UKPDS) risk engine (Kothari et al., 2002). Again, a subset of the cohort was used in order to make a more fair comparison. The subset was limited to patients newly diagnosed with type 2 diabetes (i.e. length of diabetes = 0) since the UKPDS model contains a predictor variable for length of diabetes that was not available in the current cohort. The comparison dataset was also limited to patients between 25–65 years of age and without a history of coronary heart disease. Furthermore, the subset was limited to patients with triglycerides <500 mg/dl since the total cholesterol levels used for the UKPDS prediction were calculated using the Friedewald equation which can be inaccurate in patients with extremely high triglyceride values (Warnick et al., 1990). Finally, the dataset was limited to patients who had complete data prior to imputation for the calculation of the UKPDS prediction. The final stroke comparison dataset consisted of 2,072 patients. Unfortunately, an insufficient number of events in each risk quintile prevented the creation of a calibration curve. Once again, the predictions created in this study were overfit corrected using cross-validation.

## Results and Discussion

Characteristics of patients on each of the four hypoglycemic types can be seen in Table 1. Overall the patients were predominantly white with a roughly equal gender distribution. Patients taking BIGs were younger, had less heart failure, and were more likely to have newly diagnosed diabetes. Variables included in the final models after Harrell’s model approximation (aka step-down) method are shown below in Table 2. It is important to note that the final variables do not necessarily reflect the clinical importance of that individual variable but rather the best predictive model as a whole.

**Table 1:** Characteristics of the overall cohort of patients with type 2 diabetes.

	BIG	MEG	SFU	TZD	Missing
*n*	14708	773	12606	4980	
*Continuous^a^ variables*					
Age	57.8	66.4	66.4	61.9	0
Body Mass Index	33.6	30.4	31.1	33.3	13986
Hemoglobin A1c (%)	7.8	7.1	7.7	7.8	16717
Systolic BP (mm of Hg)	133.2	133.5	135.1	133.5	6482
Diastolic BP (mm of Hg)	77.7	72.7	75	74.4	6489
LDL (mg/dl)	109.4	93.7	105.9	105.7	17347
HDL (mg/dl)	47.1	46.8	45.8	46.3	16653
Triglycerides (mg/dl)	203.7	174.8	202.5	210.6	16861
GFR (ml/min)	75.5	58	66.3	65.7	10702
Income	45047.2	43768.1	43999.7	43851.7	702
*Categorical^b^ variables*					
Male	6733 (45.8)	418 (54.1)	6961 (55.2)	2600 (52.2)	2
Caucasian	10778 (76.2)	626 (83.7)	9553 (78.2)	3917 (81.8)	1175
Heart Failure	431 (2.9)	97 (12.6)	1030 (8.2)	255 (5.1)	n/a
Coronary Heart Disesae	1528 (10.4)	147 (19.0)	1790 (14.2)	688 (13.8)	n/a
Insulin	1934 (13.2)	214 (27.7)	1371 (10.9)	1568 (31.5)	n/a
Aspirin	3566 (24.3)	243 (31.4)	3171 (25.2)	1325 (26.6)	n/a
Plavix	929 (6.3)	98 (12.7)	1059 (8.4)	516 (10.4)	n/a
ACE-I/ARB	7286 (49.5)	443 (57.3)	6699 (53.1)	2921 (58.7)	n/a
Atrial Fibrillation	476 (3.2)	50 (6.5)	757 (6.0)	191 (3.8)	n/a
Cholesterol Medication	7098 (48.3)	409 (52.9)	5630 (44.7)	2911 (58.5)	n/a
New Diabetes	3140 (21.4)	37 (4.8)	1002 (8.0)	399 (8.0)	n/a
Hypertension Medication	10242 (69.6)	639 (82.7)	9976 (79.1)	3939 (79.1)	n/a
Warfarin	858 (5.8)	120 (15.5)	1473 (11.7)	399 (8.0)	n/a
Smoking Status					8195
Never	5763 (49.0)	254 (46.7)	3941 (44.4)	1643 (44.7)	
Quit	4126 (35.1)	239 (43.9)	3649 (41.1)	1461 (39.7)	
Current	1877 (16.0)	51 (9.4)	1293 (14.6)	575 (15.6)	

**Notes.**

a Mean.

b Count (%).

BIG = Biguanide.

MEG = Meglitinide.

SFU = Sulfonylurea.

TZD = Thiazolidinedione.

BP = Blood Pressure.

LDL = Low Density Lipoprotein.

HDL = High Density Lipoprotein.

GFR = Glomerular Filtration Rate.

ACE-I/ARB = Angiotensin Converting Enzyme Inhibitor or Angiotensin Receptor Blocker.

**Table 2:** Variables included in the four final models.

	CHD	Heart failure	Stroke	Mortality
Propensity for Biguanide	Included	Included	Included	Included
Age	Included	Included	Included	Included
Gender	Included	Included	Removed	Included
Ethnicity	Included	Included	Removed	Included
Medication	Included	Included	Included	Included
Income	Included	Included	Included	Included
Glomerular Filtration Rate	Included	Included	Included	Included
Body Mass Index	Removed	Included	Included	Included
Hemoglobin A1c	Included	Included	Included	Included
Coronary Heart Disease	Not considered	Included	Included	Removed
Smoking Status	Included	Included	Included	Included
Systolic Blood Pressure	Included	Included	Included	Included
Diastolic Blood Pressure	Included	Included	Included	Included
Insulin	Included	Included	Included	Included
Clopidogrel	Included	Included	Included	Included
Aspirin	Included	Included	Included	Included
ACE-I/ARB	Included	Included	Not considered	Included
Cholesterol Medication	Included	Removed	Included	Included
New Diabetes	Included	Included	Included	Included
Low Density Lipoprotein	Included	Not considered	Included	Included
High Denisty Lipoprotein	Included	Not considered	Included	Included
Triglycerides	Removed	Not considered	Included	Included
Heart Failure	Removed	Not considered	Included	Included
Atrial Fibrillation	Not considered	Not considered	Removed	Not considered
Hypertension Medication	Not considered	Not considered	Removed	Not considered
Warfarin	Not considered	Not considered	Included	Not considered
Age X Medication	Included	Included	Included	Included
GFR X Medication	Included	Included	Included	Included
Heart Failure X Medication	Removed	Not considered	Included	Included

**Notes.**

CHD = Coronary Heart Disease.

ACE-I/ARB = Angiotensin Converting Enzyme Inhibitor or Angiotensin Receptor Blocker.

GFR = Glomerular Filtration Rate.

The final cohort sizes, median follow-up time, and model discrimination (c-statistic) are shown in Table 3, and the calibration curves appear in Fig. 1. The predictions are quite accurate across the board, with excellent calibration in CHF and CHD. The stroke and mortality models appear to be slightly less-well calibrated; mortality specifically underestimates risk in the higher risk quintiles. The concordance indices range from 0.688 to 0.753 which indicates that the model correctly identifies the higher risk patient among discordant pairs 69% to 75% of the time. An online risk calculator is available at http://rcalc.ccf.org under the heading “Type 2 Diabetes” and entitled, “Predicting 5-Year Morbidity and Mortality.” The final published calculator will be posted at http://rcalc.ccf.org.

**Table 3:** Summary of the final model results.

	Final cohort size	Median follow-up time (days)	N with Follow-up more than 5 years	C-statistic
Heart failure	25,882	503	1,211	0.7530
Coronary heart disease	23,906	462	943	0.7298
Stroke	26,140	501	1,088	0.6881
Mortality	33,067	769	1,955	0.7195

**Figure 2:**
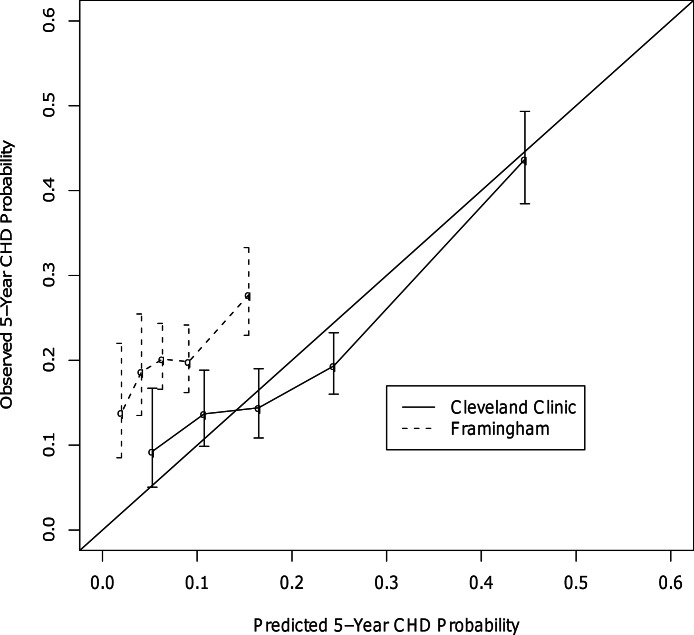
Calibration Curves for the CHD Outcome.

In the head-to-head comparisons for CHD, the final c-statistics for our CHD model and Framingham were 0.75 and 0.54, respectively. Calibration curves showed our model to be well calibrated, while the Framingham model appeared to underestimate CHD risk at all risk quintiles (Fig. 2). Other studies have also found that Framingham does not perform well in patients with type 2 diabetes (Sheridan, Pignone & Mulrow, 2003; Guzder et al., 2005; Coleman et al., 2007). Unfortunately, the CHD prediction model could not be compared head-to-head with UKPDS due to differences in the definition of CHD between the current study and the UKPDS. Specifically, the UKPDS defined CHD as MI, Fatal MI, and sudden death. It was not possible to determine which patients in the current study experienced a sudden death and it was also felt that it was important to include coronary revascularization procedures as a CHD endpoint.

For stroke, the final c-statistics were almost indistinguishable between our model (0.753) and the UKPDS model (0.752). The subset of data used for the UKPDS comparison was not large enough to create a calibration curve, but there was some evidence that the UKPDS may be underestimating stroke risk more than the stroke model created in this study when examining calibration in the large. The median predicted 5-year risk of stroke according to the UKPDS and the new model were 0.1% and 6.7%, respectively; the actual 5-year incidence of stroke was 16.4% in this subset.

The prediction calculator created in this study is the first of its kind to predict a risk profile of multiple endpoints for individual patients with type 2 diabetes. The Archimedes model has been created to estimate the overall number of different adverse outcomes in a cohort of patients with diabetes for use in cost analyses and for clinical trial simulation (Eddy & Schlessinger, 2003). But, the Archimedes model does not provide risk estimates for individual patients, and therefore cannot be compared head-to-head with the models created in this study. Other tools have been created to address individual endpoints (Framingham, UKPDS), but this tool is the first of its kind to predict multiple endpoints simultaneously for this population. This tool more closely addresses physician and patient real-world concerns for preventing a wide range of life-threatening complications. Further, it improves on the accuracy of past model’s predictions. It is interesting to note that our models created with observational EHR data matched or surpassed other published tools created using clinical trial data. Our use of propensity scores should reduce any potential concerns about treatment bias and the employment of competing risk regression should help prevent the overestimation of risks that might be caused by ignoring the competing risk of death for the CHF, CHD, and stroke outcomes.

In the past, the necessity of using a nomogram to calculate risk made large models difficult to use quickly and efficiently; replacement of this method with a free and accessible web-based calculator rectifies this issue. With the inclusion of so many individual predictor variables, the accuracy of the model is preserved while the web interface makes it easy to use. If the performance of the model is evaluated externally and found to be strong, it could be linked directly to the EHR, allowing patient characteristics to be directly imported into the calculator. All of the variables included in our models are readily accessible, making it functional for patients and clinicians everywhere.

Some limitations to our study should be highlighted. First, the duration of diabetes was not collected uniformly and therefore could not be included in the model. The patients in our cohort probably tend to be newer diabetic patients since the population was restricted to patients prescribed a single oral agent and therefore the risk calculator may be most appropriate for those types of patients. Future models may be able to out-predict ours if this variable can be captured. Of course, given the potentially lengthy period during which a patient may have undiagnosed diabetes or pre-diabetes, this variable may not add much to the accuracy of the model given the many other covariates already included. Secondly, the oral hypoglycemic included in the model was the medication at baseline. Medication changes were not considered since these future changes are unknown at the time when a new patient presents for oral hypoglycemic therapy. A final weakness of the study involves the substantial amount of missing data for some predictor variables. The imputation techniques used, however, help to limit the potential bias caused by simply eliminating incomplete records. Even with these limitations, should this model prove valid in external populations, it could prove an extremely helpful tool for clinicians who seek to understand their patient’s personal risk profile.

## Conclusions

The prediction tool created in this study was accurate in predicting 5-year morbidity and mortality among patients with type 2 diabetes. The calculator outperformed the Framingham model in predicting CHD while producing a model for stroke that had a discrimination that is comparable to the UKPDS model. The next step would be to validate this tool externally in other cohorts of patients with type 2 diabetes. If it performs well externally, it could serve as a tool for clinicians to tailor diabetes treatments to their individual patients with the aim of decreasing morbidity and improving survival.
